# Effect of biliopancreatic diversion on sleep quality and daytime sleepiness in patients with obesity and type 2 diabetes

**DOI:** 10.1590/2359-3997000000314

**Published:** 2017-12-01

**Authors:** Mayra Mello, Ana Carolina J. Vasques, José C. Pareja, Maria da S. de Oliveira, Fernanda S. Novaes, Élinton A. Chaim, Bruno Geloneze

**Affiliations:** 1 Universidade Estadual de Campinas Laboratório de Investigação em Metabolismo e Diabetes (LIMED) Campinas SP Brasil Laboratório de Investigação em Metabolismo e Diabetes (LIMED), Gastrocentro, Universidade Estadual de Campinas (Unicamp), Campinas, SP, Brasil; 2 Universidade Estadual de Campinas Faculdade de Ciências Aplicadas Limeira SP Brasil Faculdade de Ciências Aplicadas, Universidade Estadual de Campinas (FCA/Unicamp), Limeira, SP, Brasil; 3 Universidade Estadual de Campinas Unidade de Cirurgia Diabética Departamento de Cirurgia Campinas SP Brasil Departamento de Cirurgia, Unidade de Cirurgia Diabética, Universidade Estadual de Campinas (Unicamp), Campinas, SP, Brasil

**Keywords:** Bariatric surgery, type 2 diabetes mellitus, obesity, sleep, biliopancreatic diversion

## Abstract

**Objective:**

The poor quality of sleep and the deprivation thereof have been associated with disruption of metabolic homeostasis, favoring the development of obesity and type 2 diabetes (T2DM). We aimed to evaluate the influence of biliopancreatic diversion (BPD) surgery on sleep quality and excessive daytime sleepiness of obese patients with T2DM, comparing them with two control groups consisting of obese and normal weight individuals, both normal glucose tolerant.

**Subjects and methods:**

Forty-two women were divided into three groups: LeanControl (n = 11), ObeseControl (n = 13), and ObeseT2DM (n = 18). The LeanC and ObeseC groups underwent all tests and evaluations once. The ObeseT2DM underwent BPD and were reassessed after 12 months. Pittsburgh Sleep Quality Index (PSQI) and Epworth Sleepiness Scale (ESS) were applied before and 12 months after BPD.

**Results:**

Before surgery, there was less daytime sleepiness in LeanC group (p = 0.013) compared with ObeseC and T2DMObese groups. The two obese groups did not differ regarding daytime sleepiness, demonstrating that the presence of T2DM had no influence on daytime sleepiness. After surgery, the daytime sleepiness (p = 0.002) and the sleep quality (p = 0.033) improved. The score for daytime sleepiness of operated T2DMObese group became similar to LeanC and lower than ObeseC (p = 0.047).

**Conclusion:**

BPD surgery has positively influenced daytime sleepiness and sleep quality of obese patients with T2DM, leading to normalization of daytime sleepiness 12 months after surgery. These results reinforce previously identified associations between sleep, obesity and T2DM in view of the importance of sleep in metabolic homeostasis, quality of life and health.

## INTRODUCTION

The growing obesity epidemic has favored the concomitant increase in the prevalence of type 2 diabetes (T2DM) and sleep disorders (1). Partial sleep restriction decreases glucose tolerance, elevates levels of serum cortisol, reduces the release of satiety hormone leptin and increases the secretion of the hormone ghrelin, increasing hunger and appetite (2-5).

Bariatric surgery is a treatment option for obese with T2DM who does not achieve adequate metabolic control with pharmacological treatment associated with changes in life style. Studies with bariatric surgery have demonstrated improvement in sleep disorders in patients after surgery due to the impact of weight reduction (5). The biliopancreatic diversion technique (BPD) has a higher percentage of T2DM remission and sustained weight loss in the long term (6). In light of these questions, we hypothesized that massive surgical induced weight loss as observed in BPD surgery decreases the level of daytime sleepiness and improves sleep quality after long-term weight loss, as previously demonstrated in Roux-in-Y gastrectomy and sleeve gastrectomy techniques (5,7).

Given the importance of sleep in metabolic homeostasis and the association between sleep quality, obesity and T2DM, we aimed: 1) to assess daytime sleepiness and sleep quality in obese individuals with T2DM, comparing them with two control groups consisting of obese and normal weight individuals, both normal glucose tolerant (NGT); 2) to evaluate the influence of BPD surgery on sleep quality and daytime sleepiness; 3) to compare sleep quality and daytime sleepiness in operated patients with both controls.

## SUBJECTS AND METHODS

### Study design and patients

This study consisted of an experimental group and two control groups. We evaluated 42 premenopausal women who composed the following groups:

- Lean Control (LeanC): 11 normal weight (BMI = 23 ± 2 kg/m^2^) NGT;
- Obese Control (ObeseC): 13 obese (BMI = 35 ± 5 kg/m^2^) NGT;
- Surgical (T2DMObese): 18 obese (BMI = 35 ± 5 kg/m^2^) with T2DM undergoing BPD surgery.

The LeanC and ObeseC groups underwent all tests and evaluations once. The T2DMObese group was evaluated before and 12 months after surgery. At the reevaluation, two subjects did not answer the sleep questionnaires. Inclusion criteria were: age ≥ 20 years; weight variation < 5% in the last three months; negative for anti-GAD antibody; glycated hemoglobin < 10%; not taking insulin and corticosteroids; not using medications that alter sleep physiology; no liver and renal dysfunction and no recent history of neoplasia.

The protocol was approved by the Ethics Committee of the Unicamp. All participants signed the informed consent before testing.

### Clinical and biochemical evaluation

Demographics, medical history and blood pressure data were evaluated and anthropometric assessment was performed consisting of BMI, neck and waist circumferences. We evaluated body composition through the tetrapolar electrical bioimpedance technique.

The oral glucose tolerance test was performed in T2DMObese patients to confirm T2DM diagnosis (8). We collected blood samples at baseline and 120 minutes after ingestion of glucose solution containing 75 g of glucose for determination of glucose.

The levels of total cholesterol, HDL-cholesterol and triglyceride were measured by enzymatic methods. We calculated levels of LDL cholesterol by the Friedewald equation. Glycated hemoglobin was determined by high performance liquid chromatography. The blood glucose levels were measured with the Glucose Analyzer YSI 2700. The insulin was determined by chemiluminescence. Insulin resistance was assessed by HOMA-IR (9) index using measurements of blood glucose and fasting insulin.

#### Biliopancreatic diversion surgery (BPD)

The technique used is an adaptation of the original technique (10). The procedure consists of a 60% gastric resection with a *Roux-en-ϒ* reconstruction. The residual stomach volume is around 300 ml. The small intestine is transected from 280 to 320 cm from the ileocecal valve, and its distal end is anastomosed to the remaining stomach. The proximal end of the ileum is anastomosed from 80 to 120 cm away from the ileocecal valve. The total length of intestinal absorption is reduced to 280-300 cm, whose 80-120 cm are called final common channel.

#### Sleep assessment

We used the subjective evaluation method by individual application of two questionnaires: Pittsburgh Sleep Quality Index (PSQI) (11) and Epworth Sleepiness Scale (ESS) (12). The PSQI assesses the overall sleep quality for the last month (11), through 7 sleep components with weights distributed on a scale from 0 to 3, with a maximum overall score of 21. The sleep components are: subjective sleep quality, sleep latency, sleep duration, habitual sleep efficiency, sleep disorders, sleep medication use, and daytime sleepiness. The ESS assesses excessive daytime sleepiness (EDS). The score given by the respondent in all situations was summed and analyzed. A high score indicates EDS (12).

### Statistical analysis

Results were presented in median and interquartile range. The Kruskal-Wallis test was used to compare the three groups under study. The Duncan *post hoc* test was used to determine which groups differ in relation to the others. The Wilcoxon test was used to compare the T2DMObese group before and 12 months after surgery. The level of significance was p < 0.05.

## RESULTS

Clinical and metabolic characteristics at baseline and after intervention are shown in Table 1.

**Table 1:** Clinical, anthropometric and metabolic characteristics of the study groups lean control, obese control and obese type 2 diabetes

	Groups					
Characteristics	Lean Control (n = 11)	Obese Control (n = 13)	Obese T2DM Baseline (n = 18)	*p**a*	Obese T2DM Post BPD (n = 16)	*p**b*
Age (years)	29 (25 – 38)	39 (34 – 44)	46 (40 – 50)*,**	0.001	46 (40 – 50)	---
BMI (kg/m^2^)	23 (21 – 24)	33 (31 – 40)	36(33 – 39)*	0.001	28 (26 – 30)	0.001
Waist circumference (cm)	83 (79 – 85)	105 (101 – 122)	118 (107 – 125)*	0.001	98 (94 – 106)	0.001
Neck circumference (cm)	32.7 (32.7 – 33.3)	36.9 (35.5 – 38.7)	38.8 (37.3 – 40.0)*,**	0.001	35.0 (33.1 – 35.3)	0.036
Body fat (%)	29 (27 – 31)	40 (35 – 44)	42 (38 – 43)*	0.001	34 (31 – 36)	0.012
Systolic blood pressure (mmHg)	110 (100 – 115)	120 (110 – 130)	125 (110 – 140)*	0.024	120 (100 – 120)	0.010
Diastolic blood pressure (mmHg)	70 (70 – 80)	80 (70 – 90)	80 (80 – 90)*	0.080	80 (70 – 80)	0.001
HbA1c (%)	4.3 (4.1 – 4.6)	5.0 (4.5 – 5.5)	7.2 (6.5 – 8.2)*,**	0.001	5.0 (4.8 – 5.4)	0.001
Plasma glucose (mg/dL)	85 (82 – 88)	92 (89 – 97)	127 (112 – 150)*,**	0.001	91 (83 – 97)	0.001
Plasma glucose 2hPC (mg/dL)	99 (92 – 104)	120 (111 – 136)	257 (229 – 292)*,**	0.001	103 (81 – 175)	0.002
Insulin (μU/l)	5.5 (4.4 – 7.8)	7 (6 – 15.4)	13 (8.6 – 18.2)*	0.028	3.4 (2.0 – 12.0)	0.001
HOMA-IR	1.25 (0.9 – 1.62)	1.6 (1.3 – 3.5)	4.0 (2.6 – 5.5)*,**	0.002	0.60 (0.28 – 0.73)	0.001
Total cholesterol (mg/dL)	168 (153 – 184)	173 (159 – 208)	177 (169 – 201)	0.353	137 (117 – 154)	0.001
HDL cholesterol (mg/dL)	60 (50 – 66)	46 (42 – 58)	40 (34 – 44)*	0.001	43 (40 – 48)	0.015
LDL cholesterol (mg/dL)	87 (78 – 110)	105 (93 – 133)	109 (95 – 127)	0.091	62 (54 – 88)	0.001
Triglycerides (mg/dL)	57 (51 – 97)	103 (71 – 154)	124 (110-185)*	0.001	107 (75 – 142)	0.005

BMI: body mass index. Data presented as median (Interquartile range p25-p75).

a Kruskal-Wallis Test and Duncan post hoc Test (LeanC versus ObeseC versus preoperative Obese T2DM).

b Wilcoxon Test.

* p < 0.05 vs Lean C;

** p < 0.05 vs ObeseC.

**Figure 1:**
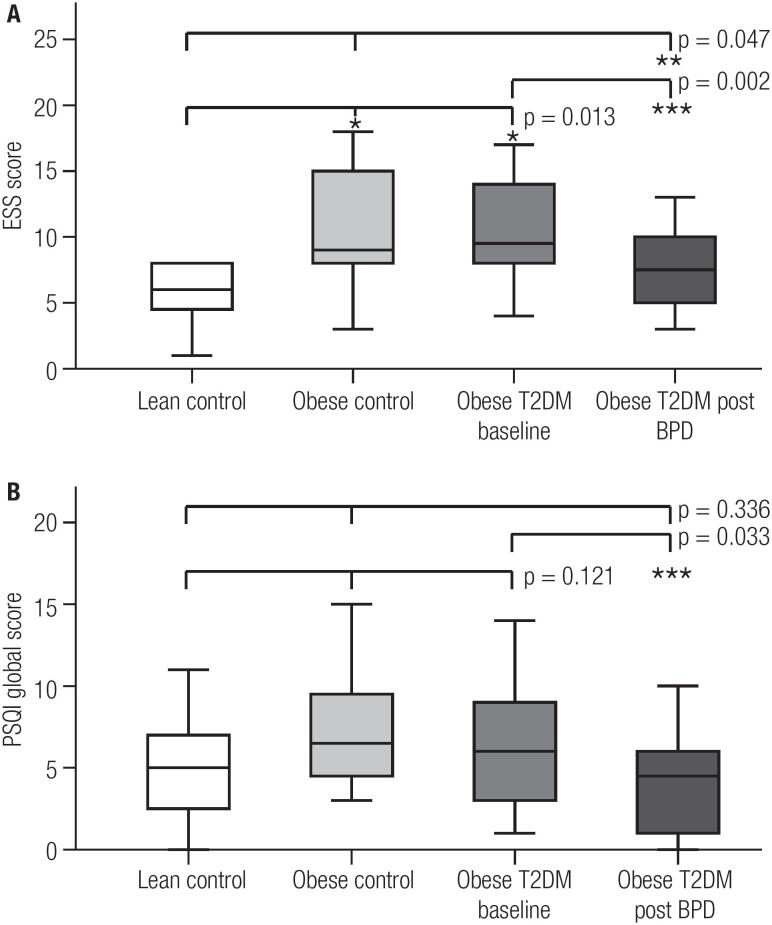
Comparison of daytime sleepiness (**A**) and sleep quality scores (**B**) in the three groups before surgery, in the surgical group pre and postoperative, and in the three groups after surgery. * p < 0.05 vs Lean Control; ** p < 0.05 vs Obese Control; *** p < 0.05 vs Obese T2DM at baseline.

Figure 1 presents the results of the subjective sleep evaluation. Before surgery, there was less daytime sleepiness in LeanC group (p = 0.013) compared with ObeseC and T2DMObese groups. The two obese groups did not differ regarding daytime sleepiness, demonstrating that the presence of T2DM had no influence on ESS (Figure 1). However, sleep quality did not differ between the three groups in the PSQI global score (p = 0.121) (Figure 1B), and also in the analysis of individual components of the score (data not shown). After surgery, T2DMObese group showed reduced daytime sleepiness and improved sleep quality. Regarding the PSQI components, the component related to sleep disorders showed significant improvement in post-BPD (p = 0.034) and there was a trend towards improvement in the component related to daytime dysfunction (p = 0.075). For the remaining components, there was no statistically significant difference. When comparing the three groups, the score for daytime sleepiness of operated T2DMObese group became similar to LeanC and lower than ObeseC (p = 0.047) (Figure 1A). For the PSQI questionnaire there was no difference in overall sleep quality (Figure 1B), although the component related to sleep disorders presented a lower score in the post-BPD patients compared with controls (p = 0.017).

## DISCUSSION

This study is the first to evaluate the effect of BPD technique on daytime sleepiness and sleep quality in obese individuals with T2DM. The main results were: 1) obese individuals, independent of T2DM presence, presented higher daytime sleepiness than normal weight patients with normal glucose tolerance; 2) BPD decreased daytime sleepiness and improved sleep quality; 3) operated subjects showed daytime sleepiness normalization, with scores similar to LeanC and reduced compared to ObeseC.

Previous studies have shown that obese individuals have worse sleep quality compared with normal weight individuals (3,5,13,14). Approximately 50% of the obese population has daytime sleepiness independent of the presence of obstructive sleep apnea. Bidulescu and cols. (13), observed higher prevalence of sleep disorders and daytime sleepiness in obese women compared with men. Another study showed that overweight is associated with worse sleep quality assessed by PSQI, especially in adult women (14). Short sleep duration is also a risk factor for obesity because of its impact on metabolism, increasing ghrelin levels and consequently appetite, resulting in weight gain; increasing evening concentrations of cortisol and whole body insulin resistance (3,15). Clinical and animal studies support the concept that obesity can directly contribute to sleepiness. The complex interrelationships between circulating systemic hormones and neuronal signaling pathways in the central nervous system are involved in this process. Obesity and metabolic syndrome are related with atypically raised basal levels of sympathetic nervous system activity, which may have the potential to fragment sleep and contribute to daytime sleepiness (16).

In this study, T2DM presence did not influence the scores for sleep quality and daytime sleepiness in obese individuals. However, studies have shown that T2DM presence is associated with worse sleep quality (17). Dixon and cols. (18), noted that the ESS scores correlated positively and significantly with elevated glucose levels. Poor sleep quality and daytime sleepiness are reported as a result of metabolic decompensation and T2DM deleterious effect on the central mechanism of breathing control. Studies have shown the influence of sleep disorders on T2DM development, making it the cause or effect of sleep disorders (15). Sleep deprivation inhibits insulin production due to elevated cortisol levels, increasing glucose levels, which can aggravate the diabetic condition (19).

Improved daytime sleepiness in patients undergoing BPD has also been observed in clinical studies with different techniques in bariatric surgery. In one of these studies, preoperative excessive daytime sleepiness was reduced from 30% to 5.5% twelve months after surgery. Patients with higher reduction in daytime sleepiness were those with more significant weight loss (20). In the present study, the surgery has improved sleep quality assessed by PSQI; however, it did not show a significant difference compared with the controls. The small number of patients studied may explain this fact, since the distribution of scores in the boxplot chart tends to decrease. The T2DMObese group showed lower score for the sleep disorder component in relation to ObeseC and similar to LeanC. A study (7) with gastric bypass and sleeve gastrectomy *Roux-en-*ϒ techniques showed sleep duration increase and consequent improvement in overall sleep quality twelve months after surgery. In this study, the improvement in sleep quality and duration was attributed to the significant weight loss (7). Jennings and cols. (21), demonstrated that the PSQI global score is positively correlated with BMI after adjustment for age among white women.

In the present study, after BPD surgery, in addition to significant weight loss, fat accumulations in the abdominal and cervical regions were reduced. Obesity results in increased deposition of fat in the neck region, including at the base of the tongue and lining the airway, especially in the throat. When the airway becomes narrowed, respiratory distress increases and may result in obstructive sleep apnea (22). In obesity, increased neck circumference has been linked to increased risk for sleep disorders (23). Our data demonstrate decreased neck circumference after surgery, which may have contributed to improved daytime sleepiness and sleep quality.

Finally, sleep loss also occurs in the presence of obstructive sleep apnea. The prevalence of obstructive sleep apnea is drastically increased in severe obesity and is around 71% in patients admitted for bariatric surgery (24). In obstructive sleep apnea patients, cortical micro- arousals are the main cause of sleep fragmentation, chronic sleep loss, and secondarily increased sympathetic nervous activity (25). The present study may have included patients with undiagnosed sleep apnea, and its resolution or improvement may be another of the mechanisms that explain the improvement in daytime sleepiness and sleep quality.

The present study has some limitations, such as: the small number of individuals in each group; the use of a single bariatric surgery technique, preventing comparisons with other techniques more used in clinical practice; the absence of a lean control group with type 2 diabetes, preventing the assessment of the isolate influence of type 2 diabetes on sleep quality and daytime sleepiness; and the absence of the polysomnography test in the study protocol.

In conclusion, BPD surgery has positively influenced daytime sleepiness and sleep quality, leading to normalization of daytime sleepiness 12 months after surgery. These results reinforce previously observed associations between sleep, obesity, and T2DM and demonstrate the beneficial effect of BPD on sleeping habits of obese patients with T2DM, in view of the importance of sleep in metabolic balance, quality of life and health.
